# Hemodynamic effects of finerenone on blood pressure and heart rate in hospitalized patients with type 2 diabetes: a real-world study

**DOI:** 10.3389/fendo.2026.1878697

**Published:** 2026-07-10

**Authors:** Song Wen, Dandan Yun, Yanju He, Xiucai Li, Lijiao Chen, Chaoxun Wang, Yulan Zhu, Dongxiang Xu, Dan Liu, Jiyu Li, Ligang Zhou

**Affiliations:** 1 Department of Endocrinology, Shanghai Pudong Hospital, Fudan University, Pudong Medical Center, Shanghai, China; 2 Fudan Zhangjiang Institute, Shanghai, China; 3 Sports-Medicine Integration Chronic Disease Rehabilitation Center, Fudan University, Pudong Medical Center, Shanghai, China; 4 Department of Rheumatology, Shanghai Pudong Hospital, Fudan University, Pudong Medical Center, Shanghai, China; 5 Department of Surgery, Shanghai Pudong Hospital, Fudan University, Pudong Medical Center, Shanghai, China

**Keywords:** albuminuria, blood pressure, finerenone, heart rate, real-world data, type 2 diabetes

## Abstract

**Background:**

While the long-term cardiorenal benefits of finerenone have been established in landmark clinical trials, its immediate impact on hemodynamic parameters in routine clinical practice remains to be fully characterized. This study aimed to evaluate the real-world acute effects of finerenone on blood pressure (BP) and heart rate (HR) in hospitalized patients with type 2 diabetes (T2D).

**Methods:**

We conducted a retrospective observational study of patients with T2D who were initiated on finerenone as part of standard clinical care. Patients were grouped into five cohorts based on the day of treatment initiation (Day 1 to Day 5). We monitored morning (a.m.) and afternoon (p.m.) systolic blood pressure (SBP), diastolic blood pressure (DBP), and heart rate (HR) daily for 7 days. Baseline characteristics, including urinary albumin-to-creatinine ratio (UACR), serum potassium, and renin-angiotensin-aldosterone system (RAAS) markers, were assessed to reflect the real-world population’s heterogeneity.

**Results:**

In this real-world cohort, finerenone was mainly prescribed to patients with notably high UACR (average grade III; p < 0.0001 compared to non-users), indicating its targeted use for high-risk kidney patients. Despite a complex background of intensive therapies—including ARBs (90.6%), CCBs (84.6%), and SGLT-2 inhibitors (91.4%)—adding finerenone was linked to significant decreases in both SBP and DBP. Daily detailed analysis showed that the largest BP reductions often occurred within the first 24–48 hours of treatment (p < 0.05 to p < 0.0001), across various treatment groups. Additionally, heart rate (HR) remained stable during the initial days of therapy. The distribution of background medications stayed consistent during finerenone treatment, suggesting that the hemodynamic improvements were not due to changes in other antihypertensive medications.

**Conclusion:**

This real-world evidence indicates that finerenone was associated with antihypertensive effects in hospitalized T2D patients with albuminuria. Its ability to substantially reduce BP and stabilize HR shortly after hospitalization, despite concurrent standard therapies, underscores its practical utility in managing hypertension.

## Introduction

Type 2 diabetes mellitus (T2DM) has become a worldwide pandemic, and its most severe microvascular complication is diabetic kidney disease (DKD) (1). Currently, DKD is the primary cause of both chronic kidney disease (CKD) and end-stage renal disease (ESRD) globally, markedly impacting the worldwide healthcare burden (2). Patients with DKD face a “dual threat”: progressive loss of renal function and a disproportionately high risk of cardiovascular morbidity and mortality (3). For decades, clinical management has relied heavily on glucose-lowering therapies and on inhibition of the renin-angiotensin system (RAS) with angiotensin-converting enzyme inhibitors (ACEIs) or angiotensin II receptor blockers (ARBs) (4). While these interventions—and the more recent addition of sodium-glucose cotransporter-2 inhibitors (SGLT-2is)—have slowed disease progression, a substantial “residual risk” persists (5). This residual risk indicates that current therapeutic strategies do not fully address the underlying drivers of cardiorenal damage, particularly the chronic inflammatory and fibrotic pathways that persist despite optimal metabolic and hemodynamic control (6).

Emerging evidence identifies pathological overactivation of the mineralocorticoid receptor (MR) as a central, independent driver of progressive kidney and heart disease (7). Traditionally, the MR was viewed primarily as a regulator of fluid and electrolyte homeostasis in epithelial cells of the renal distal tubule. However, research over the past two decades has revealed that MRs are expressed in a wide variety of non-epithelial cells, including podocytes, mesangial cells, endothelial cells, fibroblasts, and cardiomyocytes (8). In the context of T2DM, factors such as hyperglycemia, oxidative stress, and local RAS activation lead to overactivation of MR (9). This pathological signaling triggers a cascade of maladaptive responses, including the production of reactive oxygen species (ROS), activation of pro-inflammatory transcription factors such as nuclear factor-kappa B (NF-κB), and upregulation of pro-fibrotic cytokines such as transforming growth factor-beta 1 (TGF-β1) (10, 11). The resulting inflammation and fibrosis lead to podocyte depletion, thickening of the glomerular basement membrane, and interstitial scarring in the kidney, as well as myocardial hypertrophy and fibrosis in the heart (12).

The quest to block the MR pathway led to the development of steroidal mineralocorticoid receptor antagonists (MRAs), including spironolactone and eplerenone. These agents are effective in reducing mortality in patients with heart failure with reduced ejection fraction (13); Steroidal MRAs are highly lipophilic and tend to accumulate in renal tissues. Combined with their long-acting metabolites, this accumulation leads to a high incidence of life-threatening hyperkalemia, especially in patients with impaired renal function (14). Furthermore, the lack of selectivity in older MRAs often leads to anti-androgenic side effects, such as gynecomastia (15). Finerenone, a new-generation non-steroidal, highly selective MRA, has a unique chemical structure that enables a “bulkier” binding mode within the MR ligand-binding domain, providing a more balanced distribution between the heart and kidney tissues (16). Finerenone’s shorter half-life and lack of active metabolites significantly reduce the risk of potassium elevation compared with its steroidal predecessors, making it a safer and viable option for early intervention in the DKD population (17).

The clinical efficacy of finerenone was robustly established in the landmark FIDELIO-DKD trial (18), and FIGARO-DKD trials (19), which demonstrated significant reductions in composite renal and cardiovascular endpoints. There is limited data on the “acute phase” response to finerenone—the period immediately after drug initiation during hospitalization. Understanding finerenone’s performance in a real-world inpatient setting, where medications are titrated rapidly and patients may already be on intensive background therapies such as ARNIs and SGLT-2is, is critical to optimizing clinical decision-making.

Hemodynamic stability is a cornerstone of DKD management. Hypertension is both a cause and a consequence of renal damage; therefore, rapid and sustained blood pressure (BP) control is vital to protect the delicate renal microvasculature (20). Simultaneously, heart rate (HR) is a key indicator of cardiovascular autonomic tone and sympathetic nervous system activity (21, 22). In diabetic patients, elevated HR or high HR variability is strongly associated with adverse cardiovascular events and sudden death (23, 24). While finerenone is primarily recognized for its anti-fibrotic properties, its acute effects on daily BP and HR kinetic curves remain under-investigated.

This study, conducted at the Department of Endocrinology at Shanghai Pudong Hospital, was designed to address these gaps by analyzing retrospective real-world data from 462 hospitalized DKD patients. Our investigation moves beyond long-term outcomes to focus on the immediate physiological response to finerenone. By tracking daily morning and afternoon BP and HR profiles from the first day of administration through the first week, we aimed to characterize the acute hemodynamic “kinetic signature” of finerenone. Furthermore, by integrating biochemical parameters of the systemic renin-angiotensin-aldosterone system (RAAS), such as the aldosterone-to-renin ratio (ARR) and plasma renin concentration (PDC), we sought to identify the metabolic and hormonal factors that correlate with the drug’s antihypertensive efficacy. Through this real-world evidence (RWE), we hope to provide clinicians with a practical framework for the acute initiation and titration of finerenone, ultimately improving personalized care for patients with diabetic kidney disease.

## Materials and methods

### Study design and ethical considerations

This retrospective, observational study was carried out at Shanghai Pudong Hospital, Fudan University. It received approval from the Institutional Review Board (Ethics Committee) of Shanghai Pudong Hospital. As it involved retrospective analysis of de-identified data, informed consent was waived. The study adhered to the Declaration of Helsinki and local clinical guidelines.

### Patient selection and cohort definitions

We analyzed the electronic medical records (EMR) of patients admitted to the endocrinology ward between March and November 2024. 462 patients were included if they had a confirmed diagnosis of T2DM and DKD (defined by UACR ≥ 30 mg/g) and were initiated on finerenone (10 mg or 20 mg once daily) during their hospital stay. To reflect the sequential nature of real-world clinical decision-making, patients were categorized into five cohorts based on the day of finerenone initiation (Fine 1 to Fine 5). This stratification allowed us to evaluate the drug’s effects relative to the duration of hospitalization and the stabilization of other background treatments. The non-Finerenone group included patients with hypertension, whereas the Finerenone group included patients with hypertension; the only discrepancy was UACR < 30 mg/g or not exceeding DKD grade II, which is one of the treatment indications according to the medical insurance regulation in China. The observation duration for this group is identical to that of the Finerenone groups. Because they did not have detectable urinary protein on examination, the addition of finerenone is prohibited under insurance regulations.

According to established World Health Organization (WHO) and American Diabetes Association (ADA) guidelines, the included patients met the diagnostic criteria for DKD. A few severe conditions, including diabetic acidosis, hyperosmolar hyperglycemic state, lactic acidosis, shock, circulatory hypoperfusion, severe systemic disorders, stroke, asthma, uremia, intestinal obstruction, and severe sepsis, were excluded from the study. Patients with diabetes mellitus combined with primary aldosteronism, adrenal tumors, or pheochromocytoma were also excluded because these conditions could interfere with accurate assessment of Finerenone’s effects on BP and HR.

All DM patients with hypertension received a standard low-sodium diet (less than 5 g/day), along with education on medication adherence and fluid balance optimization, including correction of dehydration. They were all managed with standard glycemic control and maintained volume balance. Additionally, environmental factors and measurement times were standardized for each patient. Hospitalized patients for glycemic control and complication management received diabetes education. Their conditions remained stable, with vital signs and consciousness within normal ranges, and no signs of acute illness or complications such as diabetic ketoacidosis or hyperosmolar hyperglycemic state.

### Biochemical and clinical data collection

Baseline clinical characteristics, including age, gender, diabetes duration, and body mass index (BMI), were recorded at admission. Laboratory assessments were performed at the hospital’s central laboratory and included glycated hemoglobin (HbA1c), serum creatinine, eGFR (calculated using the CKD-EPI formula), and UACR. A comprehensive RAAS profile was established by measuring plasma aldosterone (Ald) and direct renin concentration (PDC) and calculating the aldosterone-to-renin ratio (ARR). Concomitant medications, specifically ACEIs, ARBs, ARNIs, CCBs, and SGLT-2is, were tracked daily to ensure that the observed hemodynamic changes were not confounded by abrupt adjustments to other antihypertensive or metabolic therapies.

### Hemodynamic monitoring protocol

The primary outcomes were daily changes in SBP, DBP, and HR. Hemodynamic parameters were recorded twice daily: morning (a.m., between 6:00 and 8:00) and afternoon (p.m., between 14:00 and 16:00). All measurements were obtained after at least 10 minutes of supine rest using standardized automated oscillometric BP monitors. This frequent, longitudinal data collection provided a detailed view of the immediate response to finerenone, beginning within 7 days.

### Statistical methods

All statistical analyses were performed using SPSS Statistics (Version 26.0; IBM Corp, Armonk, NY, USA) and GraphPad Prism (Version 9.0; GraphPad Software, San Diego, CA, USA). Prior to analysis, all continuous data were tested for normality using the Kolmogorov-Smirnov test. Data are presented as mean ± standard deviation (SD) for normally distributed variables, or as mean ± standard error of the mean (SEM) for longitudinal hemodynamic trends to improve visual clarity of the treatment response.

For baseline clinical characteristics (e.g., age, eGFR, UACR, and RAAS parameters), differences between the finerenone treatment groups and the non-finerenone control group were assessed using the independent-samples t-test for continuous variables and the Chi-square (chi2) test or Fisher’s exact test for categorical variables. To compare multiple treatment cohorts (Fine 1–5), a one-way analysis of variance (ANOVA) was employed, followed by Bonferroni’s *post hoc* test for multiple comparisons to identify specific intergroup differences. To characterize the acute effects of finerenone on blood pressure (SBP/DBP) and heart rate (HR), within each treatment cohort (Fine 1–5), a Repeated Measures ANOVA (RM-ANOVA) was performed. All statistical tests were two-tailed. A p-value < 0.05 was considered statistically significant. Levels of significance are denoted in the figures as follows: *p < 0.05, **p < 0.01, ***p < 0.001, and ****p < 0.0001.

## Results

### Baseline clinical characteristics and renal function

The analysis of baseline clinical characteristics revealed a highly significant disparity in the urinary albumin-to-creatinine ratio (UACR) between patients in the finerenone treatment groups and those in the non-finerenone group (p < 0.0001) **Table 1**. This finding reflects the clinical profile of patients typically selected for finerenone therapy, who often present with higher levels of protein excretion and associated renal risk. Despite the marked differences in albuminuria, other vital baseline parameters remained well balanced across all study groups. Specifically, no statistically significant differences were observed in mean age, baseline serum potassium (K+) levels, or the estimated glomerular filtration rate (eGFR) (p > 0.05). Furthermore, laboratory assessments of the renin-angiotensin-aldosterone system (RAAS)—including aldosterone (Ald) levels, direct measurement of plasma renin concentration (PDC), and the aldosterone-to-renin ratio (ARR)—showed no significant fluctuations across the groups at baseline (p > 0.05). This stability suggests that the fundamental physiological and biochemical environment was comparable across groups prior to the intervention.

**Table 1:** Baseline clinical characteristics and renal function parameters of patients.

	Age	UACR (Grade)	K+	eGFR	Ald PDC	Renin	ARR	ALD PRA	Renin	ARR
p	0.058	**<0.0001**	0.539	0.080	0.526	0.788	0.091	0.972	0.958	0.592
Day1	57.6 ± 12.7	3.4 ± 0.7	3.8 ± .0.5	81.6 ± 29.3	125.2 ± 80.0	14.5 ± 19.1	42.2 ± 98.4	55.6 ± 47.8	1.3 ± 2.3	7.7 ± 7.4
Day2	63.0 ± 12.2	3.6 ± 0.6	3.7 ± 0.5	84.3 ± 27.5	126.0 ± 105.7	22.5 ± 34.2	18.8 ± 21.2	39.4 ± 25.8	0.5 ± 0.4	14.2 ± 21.8
Day3	59.7 ± 15.3	3.3 ± 0.9	3.8 ± 0.4	78.0 ± 29.4	94.8 ± 33.9	11.6 ± 11.8	17.5 ± 21.9	41.5 ± 55.9	0.7 ± 0.7	6.6 ± 4.0
Day4	62.4 ± 15.9	3.3 ± 0.7	3.8 ± 0.7	79.4 ± 28.5	110.4 ± 68.0	27.3 ± 77.7	58.6 ± 107.4	35.6 ± 34.8	1.5 ± 1.6	1.9 ± 0.5
Day5	69.6 ± 17.9	3.4 ± 0.8	3.7 ± 0.5	75.5 ± 27.5	90.5 ± 28.0	11.1 ± 9.1	83.5 ± 185.4	51.4 ± 37.0	0.3 ± 0.1	19.6 ± 13.4
Non-Finrenone	62.9 ± 16.0	1.6 ± 1.0	3.8 ± 0.5	88.6 ± 23.7	116.1 ± 40.6	28.4 ± 77.1	22.7 ± 25.0	45.5 ± 52.5	1.2 ± 3.6	11.9 ± 16.4

Data are presented as mean ± SD.

UACR, urinary albumin-to-creatinine ratio; eGFR, estimated glomerular filtration rate; Ald, aldosterone; PDC, plasma renin concentration; ARR, aldosterone-to-renin ratio; PRA, plasma renin activity. P values indicate statistical significance across all groups. Bold values indicates p-value significant comparing via ANOVA assays.

### Dynamic trends in blood pressure and heart rate control

**Figure 1:**
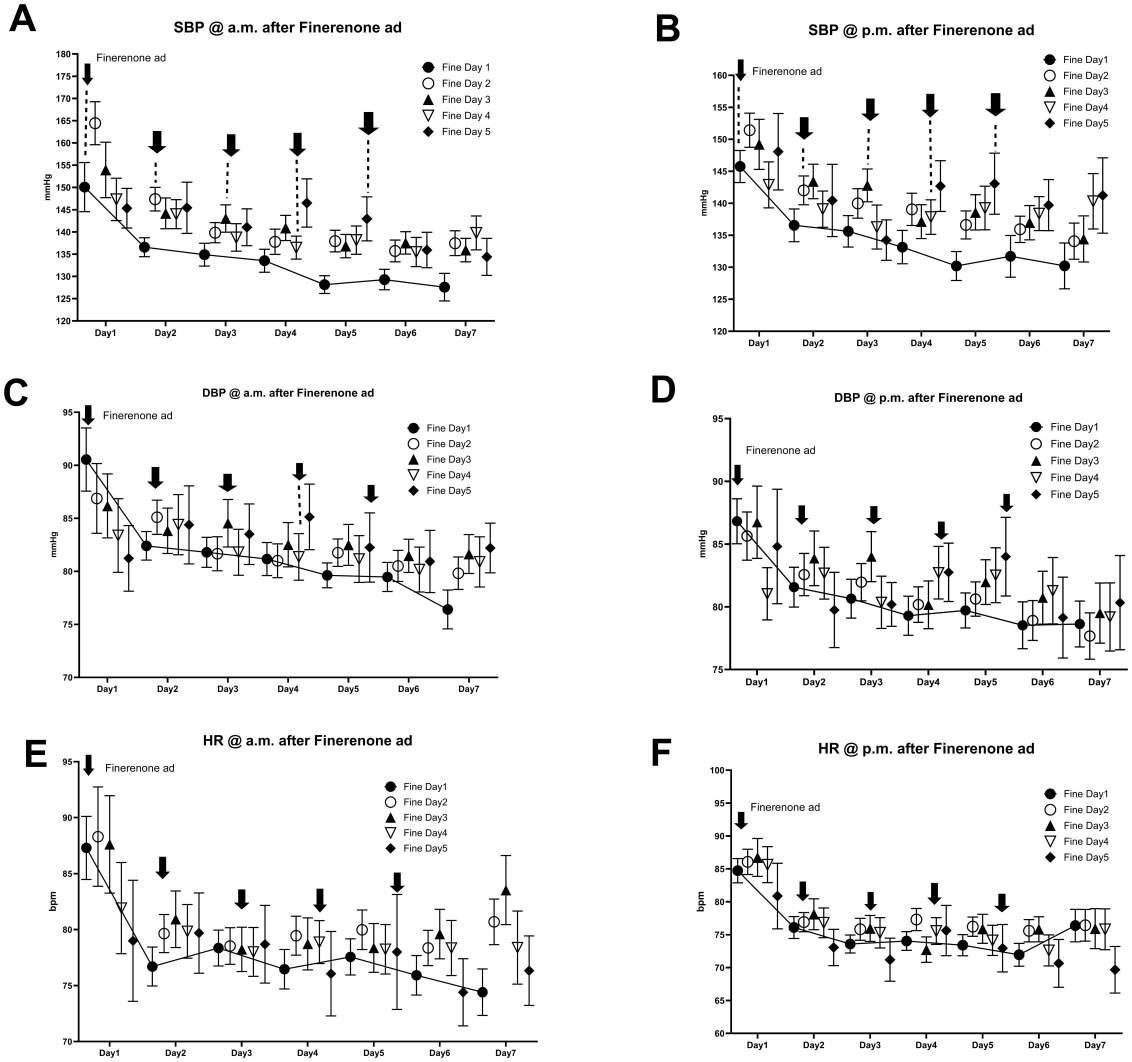
Longitudinal trends in blood pressure and heart rate following finerenone administration. **(A, B)** Morning (a.m.) and afternoon (p.m.) systolic blood pressure (SBP) trends from Day 1 to Day 7. **(C, D)** Morning (a.m.) and afternoon (p.m.) diastolic blood pressure (DBP) trends from Day 1 to Day 7. **(E, F)** Morning (a.m.) and afternoon (p.m.) heart rate (HR) trends from Day 1 to Day 7. Symbols: Different markers (circles, triangles, etc.) represent patient cohorts starting Finerenone on different days (Day 1 to Day 5). Black arrows indicate the timing of Finerenone administration.

After initiation of finerenone therapy, both systolic blood pressure (SBP) and diastolic blood pressure (DBP) showed a rapid, sustained decline throughout the hospitalization period. Longitudinal data from morning (a.m.) and afternoon (p.m.) measurements demonstrate that the antihypertensive effect began as early as the first day of administration and persisted **Figure 1**. Statistical analysis indicated that the most pronounced reductions in SBP and DBP occurred within the first 48 to 72 hours of treatment, with many of these early changes reaching high levels of statistical significance, often ranging from p < 0.05 to p < 0.0001. For instance, morning SBP in the Day 1 group showed a sharp decline that was maintained over subsequent days. In addition to blood pressure reduction, a noticeable decrease in heart rate (HR) was observed following finerenone administration. This heart rate stabilization was particularly evident in the first few days of therapy, with reductions reaching statistical significance at multiple time points, potentially suggesting a beneficial impact of finerenone on cardiovascular autonomic regulation.

### Granular daily dynamics of hemodynamic improvement

**Figure 2:**
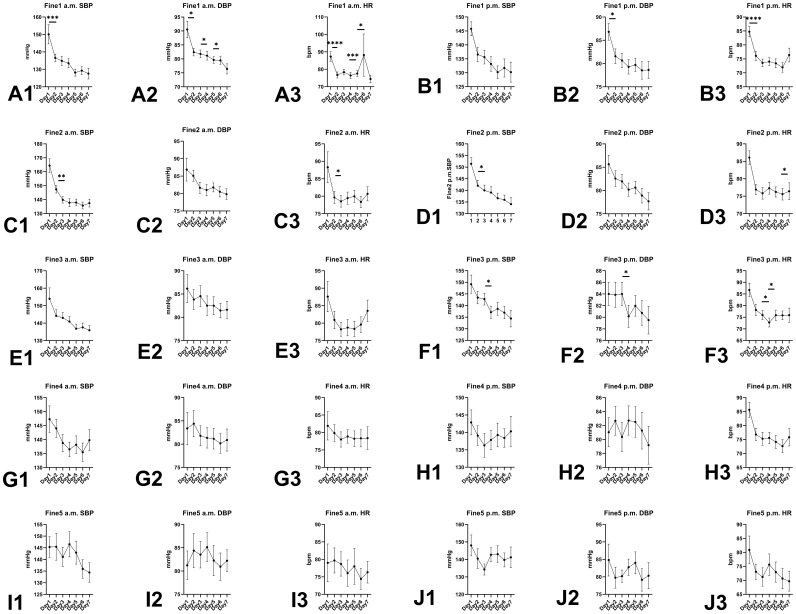
Detailed daily hemodynamic profiles for individual treatment Cohorts. **(A1–J3)** Granular daily analysis of SBP, DBP, and HR for each Finerenone treatment group (Fine 1 to Fine 5).Row 1 **(A1-B3)**: Impact of Finerenone initiation on Day 1.Row 2 **(C1-D3)**: Impact of Finerenone initiation on Day 2.Row 3 **(E1-F3)**: Impact of Finerenone initiation on Day 3.Row 4 **(G1-H3)**: Impact of Finerenone initiation on Day 4.Row 5 **(I1-J3)**: Impact of Finerenone initiation on Day 5. Statistical Significance: Significant daily changes are marked with asterisks (*p < 0.05, **p < 0.01, ***p < 0.001, ****p < 0.0001). Data are expressed as mean ± SEM.

A detailed examination of the daily subplots in **Figure 2** reveals a consistent pattern of rapid hemodynamic improvement immediately after Finerenone initiation across cohorts. In the Fine 1 cohort (patients starting on Day 1), a sharp, statistically significant drop in morning SBP was observed between Day 1 and Day 2 (p < 0.001, **Figure 2-A1**), a trend mirrored in the afternoon recordings (p < 0.05, **Figure 2-B1**). This immediate response was similarly evident in the Fine 2 and Fine 3 cohorts, with significant reductions in both SBP and DBP within the first 24–48 hours of drug administration (**Figures 2-C1, D1, F1, F2**). Notably, heart rate (HR) also showed significant acute deceleration; for example, the Fine 1 a.m. HR dropped markedly from Day 1 to Day 2 (p < 0.0001, **Figure 2-A3**). While the magnitude of the reduction was most pronounced and statistically robust in the cohort’s starting treatment in the first three days (Fine 1–3), those starting later (Fine 4–5) continued to show a general downward trend in blood pressure, albeit with increased variability as the study approached Day 7 (**Figures 2-G1, J3**). These granular findings confirm that Finerenone exerts a rapid antihypertensive effect detectable within the first 24 hours of therapy.

### Evaluation of concomitant antihypertensive and metabolic therapies

The study also conducted a comprehensive evaluation of background therapies to ensure that the observed outcomes were attributable to the study drug **Table 2**. At baseline, the finerenone group had a significantly higher utilization rate of several classes of medications, including Angiotensin II Receptor Blockers (ARBs), Calcium Channel Blockers (CCBs), Angiotensin Receptor-Neprilysin Inhibitors (ARNI), and Sodium-Glucose Cotransporter-2 (SGLT-2) inhibitors, compared to the non-finerenone group (p<0.05). However, during the critical first three days of finerenone administration (Day 1–3), the use of core antihypertensive agents such as ACE inhibitors, ARBs, ARNIs, and β-blockers remained remarkably stable with no significant changes in dosage or frequency (p > 0.05). This stability effectively eliminates the possibility that the observed drop in blood pressure was caused by adjustments to background medications, thereby confirming that the antihypertensive gains were primarily driven by the addition of finerenone. While a slight variation in CCB usage was noted by Day 4 and Day 5 (p = 0.032), the overall therapeutic regimen for most antihypertensive and metabolic classes remained consistent throughout the acute phase of the study.

**Table 2:** Concomitant antihypertensive and metabolic medications.

Anti-hypertensive drug	ACEI	ARB	ARNI	CCB	αBlockade	βBlockade	Diuretic	Loop	SGLT-2
Non-Fine (n=248)	12(4.8%)	87(35.1%)	1(0.40%)	77(31.0%)	6(2.4%)	27(10.9%)	23 (9.27%)	12(4.8%)	96(38.7%)
Finerenone (n=195)	7(3.6%)	106(54.4%)	13(6.7%)	99(50.1%)	22(11.3%)	19(9.7%)	33(16.9%)	25(12.8%)	107(54.9%)
P value	0.948	0.001**	0.001**	0.0001****	0.001***	0.875	0.079	0.031*	0.012**
Finerenone									
Day 1 ad (n=57)	3 (5.3%)	29(50.9%)	4(7.0%)	24(42.1%)	6(10.5%)	5(8.8%)	9(15.8%)	8(14.0%)	27(47.4%)
Day 2 ad (n=58)	3(5.2%)	34(58.6%)	5(8.6%)	34(58.6%)	8(13.8%)	5(8.6%)	13(22.4%)	9(15.5%)	35(60.3%)
Day 3 ad (n=39)	1(2.6%)	21(53.8%)	3(7.7%)	21(53.8%)	5(12.8%)	4(10.3%)	4(10.3%)	4(10.3%)	17(43.6%)
P value (Day1-3)	0.799	0.725	>0.05	0.196	0.867	>0.05	0.3	0.796	0.367
Day 4 ad (n=25)	0(0%)	11(44%)	1(4%)	8*(32%)	2(8%)	2(8%)	4(16%)	2(8%)	18(72%)
Day 5 ad (n=16)	0(0%)	11(68.8%)	0(0%)	12*(75%)	1(6.3%)	3(18.8%)	3(18.8%)	2(12.5%)	10(62.5%)
P value (All Day)	0.862	0.553	0.912	**0.032**	0.936	0.767	0.637	0.911	0.058

Values represent the number of patients receiving each medication class. ACEI, angiotensin-converting enzyme inhibitor; ARB, angiotensin II receptor blocker; ARNI, angiotensin receptor-neprilysin inhibitor; CCB, calcium channel blocker; SGLT-2, sodium-glucose cotransporter-2 inhibitor3.

Significant differences (p < 0.05) are indicated by asterisks. Bold values indicates *: p<0.05, **: p<0.01; ***: p<0.001; ****: p<0.0001.

## Discussion

The present study provides robust Real-World Evidence (RWE) demonstrating that finerenone, a novel non-steroidal mineralocorticoid receptor antagonist (MRA), exerts an antihypertensive effect in hospitalized patients with diabetic kidney disease (DKD). Our granular analysis, specifically 7-day longitudinal daily monitoring across five sequential initiation cohorts (Fine 1–5), shows that significant reductions in systolic blood pressure (SBP) and diastolic blood pressure (DBP) occur as early as 24–48 hours after administration.

In large-scale clinical trials such as FIDELIO-DKD, the long-term renal and cardiovascular benefits of finerenone are well documented; however, its acute hemodynamic “kinetic signature” has remained largely under-investigated (18, 19). Unlike steroidal MRAs such as spironolactone, which have a delayed onset of action due to their lipophilic pharmacokinetic profiles and long-acting active metabolites, finerenone’s non-steroidal structure and specific binding affinity enable near-immediate receptor modulation (13, 15).

Nowadays, non-invasive techniques are emerging for evaluating microvascular function and its responses to acute physiological and pathological stressors (20). In parallel, preclinical evidence indicates that hemodynamic modulation could be regulated by SGLT-2i, which activates central nervous system neurons to regulate the sympathetic neural pathway (21), along with a structured summary of the central mechanism of SGLT-2i (22), suggesting it may share finerenone’s basal mechanism, which could antagonize RAAS activation. A recent epidemic study also indicated that microvascular complications caused by diabetes could increase the prevalence of heart failure (HF), a macrovascular complication (23). Novel antidiabetic medications, such as SGLT-2i, disclosed in a recent meta-analysis, not only prevent HF but also reduce microvascular complications, including DKD, as indicated by reductions in UACR and eGFR (24). Collectively, the early hemodynamic signature identified in our hospitalized cohorts, when integrated with finerenone’s pleiotropic and largely blood pressure-independent actions, highlights its distinctive therapeutic profile in DKD that extends beyond conventional antihypertensive mechanisms. For hospitalized patients, this rapid onset is of paramount clinical importance because it enables swift stabilization of intraglomerular pressure, potentially halting the mechanical drivers of podocyte injury during the acute phase of admission (25).

Our prior research established that an imbalanced aldosterone-to-renin ratio (ARR) is a significant independent predictor of renal microvascular disease, even when systemic aldosterone levels remain within the laboratory’s “normal range” (26). The imbalanced ARR indicates relative aldosterone excess, which drives chronic mineralocorticoid receptor (MR) overactivation, leading to oxidative stress and tissue remodeling (27).

In the current cohort, the rapid antihypertensive response to finerenone was particularly pronounced among patients with elevated baseline ARR. This clinical observation directly supports the mechanistic hypothesis that finerenone works by effectively intercepting pathological MR signaling that is disproportionately high in patients with salt-sensitive or aldosterone-driven hypertension (28). By antagonizing the MR, finerenone not only lowers systemic blood pressure but also specifically reverses the pro-inflammatory and pro-fibrotic milieus characterized by NF-κB activation and TGF-β1 upregulation (6, 10). This mechanism bridges the gap between the biochemical findings in our previous work and the clinical outcomes observed here, suggesting that ARR could serve as a vital real-world biomarker for predicting the magnitude of finerenone’s hemodynamic and renal benefits.

In our study, over 91% of patients were already receiving SGLT-2 inhibitors, and over 90% were on RAS inhibitors (ARBs or ARNIs). Persistent residual risk in DKD management is often attributed to “aldosterone escape” or insufficient blockade of inflammatory pathways despite standard-of-care (29, 30). Additionally, although we added Finerenone to treat proteinuria on the 4th and 5th days in patients whose BP and HR were lower than in the other 3 groups, we did not observe significant BP and HR fluctuations, even among those treated only with ACEI, ARB, or CCB, suggesting that this antihypertensive effect was dependent on the baseline BP and HR.

This synergistic effect can be explained by the complementary action of SGLT-2 inhibitors and nonsteroidal MRAs (31). SGLT-2 inhibitors primarily modulate metabolic pathways and reduce proximal tubular sodium reabsorption, while finerenone targets the distal nephron and vascular MRs to counteract fibrosis. Importantly, the stability of background medications during the first 72 hours of our study—as evidenced by the lack of significant changes in ACEI/ARB/CCB dosages—isolates finerenone as the primary driver of the observed acute hemodynamic improvements. This confirms that finerenone is a powerful “add-on” tool that functions independently of, yet synergistically with, current regimens.

An intriguing finding in this real-world cohort was the stabilization and significant reduction in heart rate (HR) alongside blood pressure lowering. None of the finerenone cohorts exhibited reflex tachycardia, a common side effect of many vasodilators; instead, many patients showed a downward trend in HR (32). This suggests that finerenone may modulate cardiovascular autonomic tone.

Patients with T2DM and DKD frequently develop cardiovascular autonomic neuropathy (CAN), characterized by sympathetic overdrive and elevated resting HR. MRs located in the central nervous system and on vascular baroreceptors are known to influence sympathetic outflow (33). By inhibiting these receptors, finerenone may improve baroreflex sensitivity and suppress the overactive sympathetic system (34). Given that elevated HR is an independent predictor of sudden cardiac death and heart failure hospitalization in diabetic populations, this dual “BP-lowering and HR-stabilizing” effect offers a more comprehensive cardiorenal protective profile than previously recognized.

Within the Cardiorenal Metabolic Syndrome (CKM) framework, HbA1c-based metabolic stratification directly dictates systemic biological heterogeneity and early glucotoxicity. In early-onset metabolic syndrome, premature glucotoxicity and lipid dysregulation accelerate chronic low-grade inflammation and oxidative stress (35). This microenvironmental stress directly compromises the glomerular filtration barrier, driving early podocyte injury and proteinuria—the definitive hallmarks of subclinical CKM progression. Although over 91% of our cohort received background SGLT-2i therapy to modulate proximal tubular adaptations, the persistent residual risk underscores that metabolic control alone cannot halt autonomous tissue inflammation. Adding finerenone to this metabolically stratified population precisely targets downstream MR hyperactivation amplified by early-onset metabolic stress, thereby dampening systemic inflammatory pathways, mitigating early proteinuria, and stabilizing hemodynamic curves before irreversible multi-organ CKM damage occurs.

This neurohormonal stabilization is clinically vital for the primary prevention of early-onset stroke in patients with complex underlying vascular pathologies. Hyperaldosteronism, signaled by an elevated baseline ARR, acts as a primary hidden driver of maladaptive vascular remodeling, intimal hyperplasia, and smooth muscle cell hypertrophy. When this mineralocorticoid-driven vascular stiffness intersects with fragile, complex cerebral angiopathies—such as Moyamoya disease (MMD), characterized by terminal carotid stenosis and network-like collateral formation—the risk of acute cerebrovascular accidents escalates exponentially (36).

In these vulnerable populations, early-onset stroke is easily triggered either by hypoperfusion-induced ischemia or by hemorrhagic rupture of fragile collateral vessels during abrupt blood pressure surges. Finerenone’s acute kinetic signature—characterized by a rapid, smooth, and reflex-tachycardia-free pressure reduction—provides a critical dual defense: it mitigates mechanical shear stress on compromised arterial walls while its potent inhibition of aldosterone-mediated structural remodeling and chronic inflammation directly stabilizes both systemic nephrons and cerebral microvascular beds. Counteracting the destructive synergy of high ARR and premature metabolic syndrome with non-steroidal MRAs shifts the clinical paradigm from reactive crisis management to the primary prevention of chronic inflammatory injury and premature cerebrovascular mortality.

For frontline clinicians, these findings suggest that initiating finerenone in a hospitalized setting is both safe and effective for rapid hemodynamic optimization. The high-resolution, daily EMR-based monitoring used in this study provides a level of detail often missing from outpatient trials. Although the retrospective nature of this RWE study has limitations, it reflects the true complexity of DKD management and underscores finerenone’s role as a cornerstone of modern cardiorenal therapy.

### Limitations

We acknowledge several limitations. As a retrospective observational study, it is subject to selection bias in the timing of finerenone initiation. Additionally, although we observed significant acute benefits, the 7-day inpatient observation period is insufficient to draw conclusions about long-term changes in hard endpoints such as ESRD or major adverse cardiovascular events (MACE). Future prospective studies should investigate whether the magnitude of this “acute phase” hemodynamic response can predict long-term renal survival.

## Conclusion

In summary, this study provides critical evidence that finerenone administration in hospitalized DKD patients was associated with the alleviation of uncontrolled hypertension. By effectively reducing BP and HR within 48 hours, especially in patients with an imbalanced ARR, finerenone addresses the MR-mediated residual risk that persists despite standard therapy. Future studies will examine the long-term practical utility of finerenone as a potent, safe, and synergistic add-on therapy, and its role in the acute and chronic management of diabetic kidney disease.

## Data Availability

The datasets presented in this article are not readily available because the availability of data in this study could be requested on contacting of corresponding author. Requests to access the datasets should be directed to zhouligang1n1@163.com.
